# Strictures of the Cervical Canal, and of the Internal and External Os

**Published:** 1878-08

**Authors:** Fredrik Eklund

**Affiliations:** Stockholm, Sweden, Member of the Swedish Society of Physicians; Physician of the First Class in the Swedish Royal Navy; Foreign Honorary Graduate of the Medical Department of the University of Georgia, etc., etc.


ATLANTA
### Medical and Surgical Journal.

VOL. XVI.] AUGUST—1878. [No. 5

### Original Communications.

### STRICTURES OF THE CERVICAL CANAL AND OF THE INTERNAL AND EXTERNAL OS.

BY FREDRIK EKLUND, M.D., STOCKHOLM, SWEDEN,
Member of the Swedish Society of Physicians; Physician of the First Class in the Swedish Royal Navy; Foreign Honorary Graduate of the Medical Department of the University of Georgia, etc., etc.

TRANSLATED FROM THE SWEDISH,
As Originally Published in the "Nordiskt Medicinskt Arkiv," Band. VIII., Heft. 3.,
BY
A. SIBLEY CAMPBELL, A.B.; M.D., AUGUSTA, GA.

### TRANSLATOR'S PREFACE.

In presenting a translation in full of Dr. Eklund's admirable monograph, I would invite especial attention to his views as to the etiological bearings of the question, and to his excellent classification—more complete, I believe, than is to be found in any other treatise on the subject.

Growing out of his views as to etiology, the indications as to prophylaxis assume a position of prime importance, challenging our earnest study and attention. The divisions of the question which relate to pathological anatomy, diagnosis, prognosis and treatment, are also carefully considered;—the whole being well illustrated by a sufficient number of selected cases, accurately reported and arranged under the different classes to which they belong.

Without anticipating further by any prefatory statement in regard to the author's deductions and the arguments upon which they are founded, I will only express the hope that justice has been done the original in the attempt thus to render it in its present form.

### ETIOLOGY.

During the continuous progressive development of science, it is inevitable that conflicts of opinion will arise in regard to a multitude of different questions. If now these antagonistic views relate only to subjects of trivial weight, it is of little importance if we pass them over without notice. Should, on the contrary, an opposite condition exist, and in questions which are of the greatest moment, men of great distinction in the profession express opinions which, notwithstanding repeated protests, they persistently maintain, every one must feel himself called upon to record his experience; so that, through the many-sided lighting of the question, he may contribute to the advancement of the truth. Among such questions of the greatest weight and interest should be reckoned the present teachings in regard to strictures of the cervical canal and of the orifices of the uterus.

Without the fear of being contradicted, I venture to maintain that the disorders under consideration, taken in the proper sense, by analogy with strictures of the urinary canal in the male (excluding here any question as to such strictures), were in this country until a short time ago exceedingly rare or at least little noticed. In the circular of the Royal Sanitary College, issued to all physicians in the kingdom, with a revised formula for the sick-estimates and reports, and with also a reformed nomenclature for the purpose of recording the various diseases—published in Stockholm on the 31st of August, 1874—in which strictures of the small intestine, of the rectum, of the œsophagus, and of the urethra are found recorded, we fail to find strictures of the cervical canal and of the orifices of the uterus. So far, I have not known even a single case of the disease last named to have been announced in the public hospital reports, and from private practice only two[1]. The same condition of things existed until a short time ago in Great Britain, where according to W. CUMMING[2] real strictures of the cervix uteri were extremely rare (although indeed not so rare as strictures of the male urethra in subjects who had not been affected with gonorrhœa); whereas, incision of the cervix or hysterotomy for the cure of strictures, was not at all a rare operation in the last named country. M. DUNCAN[3] also expresses himself to the same effect, having never seen or heard of strictures, in the ordinary surgical sense, in the cervical canal or the orifices of the uterus.

But besides the fact that my comparatively limited experience gives support to the supposition that with us the frequency of these disorders is involved in doubt, information derived from other sources comes to confirm the opinion that the condition of things is one and the same in other countries. Surprising indeed is the report of ED. MARTIN[4], according to whom three hundred and eighty-six operations for stenosis of the external os and cervical canal were performed by himself since 1850. Likewise HEGAR and KALTENBACH[5] seem to have an extensive experience in regard to the troubles in question, as they state that out of more than one hundred and fifty discissions they have only two deaths to lament[6].

---

1 Sven Skoeldberg: Nordiskt Medicinskt Arkiv. Band. I., N:r. 9., 1869, p. 6.

2 The Retrospect of Medicine. Edited by W. and J. Braithwaite; July–Dec. 1873, pp. 305–6.

3 Ibid., Vol. LXVII., Jan.–July 1873, p. 301.

4 Zeitschrift fur Geburtskunde und Frauenkrankheiten. Band I., Heft 1., 1875, p. 115.

5 Die Operative Gynækologie. Von Dr. A. Hegar und Dr. R. Kaltenbach. Erlangen; 1874, p. 292.

6 [In regard to the operation of incision and division of the cervix, Dr. Sims says: "Since 1856 I have operated many hundred times, nearly a thousand, not always for dymenorrhœa, and I have lost two patients by it—one in 1869, the other in 1873." Dr. Pallen states that he has operated for stenosis of the cervical canal 337 times since Nov. 1865, with two deaths. Pallen: Incision and Division of the Cervix Uteri for Dysmenorrhœa and Sterility. American Journal of Obstetrics, Vol. X., No. 3. pp. 375 and 386.]—TRANSLATOR.

More than a decade past, OPPOLZER urged that medicine must pass over from the pathologico-anatomical standpoint, and take her stand upon the etiological. More than once he expressed his fully confident hopes that, with a reformation in the direction just mentioned, health bringing fruit would ripen to harvest for suffering humanity. OPPOLZER's eloquent voice is hushed, but the truths which he proclaimed outlive him, and are now unfolded in their excellence by his heedful and faithful disciples.

And so in regard to stenosis of the cervical canal and of the orifices of the uterus, shall a clearer insight into the causes of these disorders bring it to pass that, even if they are not entirely eradicated, their number shall yet be reduced to a minimum. But before I state my observations and enter into an analysis of them, permit me to enter upon a few general considerations.

A priori, it must be admitted, that by analogy with the processes which take place in regard to other canals in the organism, after all diseased conditions which are attended with neoplasms of the connective tissue, or after solutions of continuity, which are healed with a gradually contracting cicatrix, a stricture at some point must generally result. SCHROEDER[1] also regards the causes which produce stenosis of the cervix uteri to be of the most varied nature: "they may thus arise when the calibre of the canal becomes closed up through inflammation and engorgement, and further from inflammation of the mucous membrane when at the same time the engorged cervical follicles (ovula Nabothi) are ruptured, and their granulating walls become adherent to each other; and besides these causes, from cicatricial formations of all kinds:" of these he makes special mention only of those which are produced by cauterization; but foremost among all the causes he regards injuries during parturition and the puerperal inflammatory processes. In particular is this opinion of SCHROEDER's shared by E. MARTIN[2] who re-

1 Handbuch der Speciellen Pathologie und Therapie. Herausgegeben von Dr. H. von Ziemssen. Band X. Krankheiten der Weiblichen-Geschlechtsorgane, von Prof. Carl Schrœder, in Erlangen. Leipzig 1874, p. 61.

[See, also, American Edition, p. 66.]—TRANS.

2 E. Martin, op. cit., pp. 108–9 et seq.

gards these strictures in the great majority of cases to be "acquired, and to depend upon deep spontaneous ulcerative lesions during pregnancy;" but he believes they chiefly originate in puerperæ, for instance from diphtheritic formations, or in consequence of vaginal injections with caustic liquids, e. g. sulphuric acid; and in the non-gravid from contracting exudations after inflammations of the inner margin of the lips of the uterus with or without ulceration, to which he believes repeated cauterizations with nitrate of silver in substance contributes; whereas, he scarcely in a single instance observed this result after numerous cauterizations with liquor nitratis hydrargyri. As these authors hold, it is consequently a variety of different causes which may produce the same result, namely stenosis; but it is plain, that in a purely practical respect the essential weight rests upon the correct answer to the question: What are the most commonly observed causes, or at least those which in the majority of cases, or with great certainty, produce the strictures in question, and what are the causes which only very rarely make their influence felt? As the future weal or woe of many depends upon a true answer to this question, perhaps every contribution to its solution, even though insignificant, will be welcome; and to afford it such assistance, is the chief aim of this paper.

Martin[1] answers the question in the following manner: "In the majority of cases stenosis arises in sterile young wives from gonorrhœa in the husbands, although I do not deny the possibility that scrofula, or that various kinds of mechanical irritation, such as intra-vaginal self-abuse, may under certain conditions produce an inflammation which gives origin to stricture. Comparatively more rarely does Martin consider that the diseased conditions above mentioned, (p. 4) are observed as the causes of stenosis.

Touching the great weight which Martin places upon gonorrhœa in the male as the cause of stricture of the cervical canal in sterile young wives, the same views have

1 E. Martin, op. cit., p. 110.

been expressed by the same writer[1] now for more than eleven years past. But MARTIN at once encountered strong opposition. Besides, G. BRAUN[2] next declared that he could allow Martin's proposition to have current force in only a few cases. It deserves to be remarked further, on the question as to blennorrhœa as the cause of strictures of the cervical canal, that BERNUTZ and GOUPIL[3] long ago considered themselves authorized—on the ground of a sufficiently large number of cases—to deliver the opinion that none of the women affected with blennorrhœa, whom they saw, had exhibited adhesions, or cicatrices of the vagina, nor any constriction of the cervical canal; a few, however, as the result of blennorrhœa, had been the prey to dysmenorrhœal sufferings, which they formerly did not at all experience; but in these cases the blennorrhœa had produced a pelvi-peritonitis and consecutive uterine deviation (?) or it had left behind as a sequel, a uterine catarrh, to which they believed they could attribute the disorder in the excretion. The prime resultant of their opinions is, that strictures as sequelæ of venereal affections—be it gonorrhœa or syphilis—are entirely excluded; at least, when in conformity with the authors mentioned, we abstain from cauterization.

The standpoint, on which F. A. SIMON[4] placed himself, when he expressed the opinion, that with gonorrhœa the mucous membrane of the cervical canal and of the uterus itself is very seldom concerned, must now be regarded as altogether overthrown. Without doubt BARNES[5] has already come much nearer the truth, when he maintains that blennorrhœa has a great tendency to invade the cervical canal and afterwards to become chronic; and accord-

---

1 Monatsschrift fur Geburtskunde und Frauenkrankheiten. Band XXV., p. 107.

2 Medizinische Jahrbucher. Redigirt von C. Braun, A. Duchek und L. Schlager. Band XI., p. 98.

3 Clinique Medicale sur les Maladies des Femmes. Par G. Bernutz et E. Goupil. Paris, 1860. Tome I., p. 87.

4 Handbuch der Speciellen Pathologie und Therapie. Band II., Abtheilung I: Syfilis; von F. A. Simon, p. 501.

5 R. Barnes: Clinical History of the Diseases of Women. London, 1873, p. 856.

ing to my opinion BAUMES[1] is perfectly correct in this, that with blennorrhœa the uterus and tubes are invaded by degrees, and also that the disease there becomes latent[2] or extends thence to the ovaries. It is generally known that most cases of gonorrhœa in men return to health without being followed by strictures; and especially if we refrain from employing the so-called abortive agents (nitrate of silver and other caustics in concentrated form) for destroying the contagion. Cases get well in which, through these agents, a rapid cure is accomplished; but on the other hand we have also very often observed strictures after the employment of concentrated caustics.[3]

Strictures occurring in men as a consequence of gonorrhœa, have their seat principally in the membranous portion, it being the narrowest part of the urethra.[4] In agreement therewith they occur in women, who are nulliparæ, most often in the lower portion of the cervical canal, which in these has a spindle shape; and in women who have borne children, in the upper portion of the canal, which as the result of puerperal involution, is generally narrowest at this point. Yet it is entirely undemonstrated, that blennorrhœa itself is the essentially originating cause, so long as strictures of the cervical canal and the orifices of the uterus constitute in prostitutes—the majority of whom are the most affected with blenorrhœa—a very rare or at least little noticed disease. And if now in those women who are pre-eminently the subjects of blennorrhœa, and very often to a particularly intense degree, so extremely seldom strictures are observed, how

---

1 Handbuch der Speciellen Pathologie und Therapie, op. cit., loc. cit.

2 Nœggerath. [Emil Nœggerath: Die Latente Gonorrhœ im Weiblichen Geschlecht; Bonn, 1872. Also, "Latent Gonorrhœa especially with regard to its influence on Fertility in Women." Transactions American Gynæcological Society. Vol. I., 1876, p. 268.]—TRANS.

3 Compendium der Chirurgischen Pathologie und Therapie. Von Dr. C. Heitzmann. Wien, 1869, p. 128.

4 Grævell's Notizen fur Praktische Arzte. Berlin, 1873, p. 596, (Rabisch's review). Of 378 cases, (of which 204 were produced by blennorrhœa), which during the years 1862–1872 were observed at the public hospital in Vienna, 118 had their seat in the pars membranacea, 15 in the pars spongiosa, 3 in the pars prostatica, 10 in the pars membranacea and pars spongiosa, and 29 in the bulb.

much less then should we ascribe to gonorrhœa in the male the power to produce these unfortunate results, even admitting—though still far from being according to the rule, but only exceptionally—that from a gonorrhœa in the husband originates an acute blennorrhœa in the wife. Every physician of any experience on this subject quite certainly knows various patients, married as well as single, who for ten, twenty or thirty years past, evidently had blennorrhœa without stricture occurring in the cervical canal.

If, consequently, we cannot adjudicate to gonorrhœa in the male, as a cause of stricture of the cervical canal, any specially great significance, there still remains the fact that constrictions in the canal of the neck of the uterus are each year more and more frequently coming under treatment. In order to be able in a satisfactory manner to solve our problem, we must now turn our attention in another direction; and nothing then suits our purpose better than a consideration of the diseases which most commonly occur in the cervix uteri, and the methods which are principally and most frequently employed for their relief.

Of these diseased conditions, catarrh—the usually faithful attendant of derangement—occupies, in regard to its frequency, the most prominent position, and exhibits different intensities, from simple catarrh, through the intermediate erosive and ulcerative catarrhs, up to those severer forms, granular (vegetating and fungous) catarrhs, which are characterized by hypertrophy of the papillæ observed below, and neoplasms of the like higher up in the cervical canal. LINDGREN[1] has found that in children a few months old no papillæ appear in the mucous membrane of the cervical canal. In the adult, according to the same author, it is far from being the case that papillæ constantly appear in the mucous membrane of the cervical canal; that they are usually not observed in the perfectly healthy virgin uterus; and that they appear developed and numerous in proportion as the uterus becomes

1. Nordiskt Medicinskt Arkiv. Band III., N:r. 13. Hj. Lindgren: Om Lifmoderns Byggnad, pp. 28-9.

exposed to irritation of any kind; consequently, especially in older women, in whom he not rarely met with free papillæ. According to HENLE[1] papillæ are found only in the lower portion, with a thicker layer of pavement epithelium covering that portion of the cervical canal. No one denies that the milder forms of uterine catarrh, even in conjunction with erosions, may recover under a proper course of treatment directed merely against the general derangement; likewise, that the ulcerations so often observed are usually so superficial and insignificant that they are healed without cicatricial contraction, if we only refrain from powerful caustics; whereas, the more extensive ulcerations and granulations demand local treatment, in which we must determine to employ such agents as shall counteract if possible the tendency to cicatricial contractions, which at least the severest forms of the pathological condition last mentioned necessarily occasion. For effecting a cure of these, the most varied caustic and astringent agents have been employed, from the ferrum candens, advised by JOBERT,[2] down to tannin, which is recommended and employed with success in crayons, for uterine catarrh, by Prof. A. ANDERSON.[3]

As to the former of these measures, its employment can probably only be taken into consideration in the very severest cases; with regard to the tannin treatment, the name of the distinguished teacher constitutes a certain safeguard as to the reliability of the agent. The following agents have been separately proposed as efficacious in the diseases under consideration: namely, the crayons of sulphate of zinc by BRAXTON HICKS, zinc-alum crayons by SVEN SKŒLDBERG, nitrate of silver by BARNES and others, solid Vienna paste by FILHOS, liquor nitratis hydrargyri, employed for the first time in the latter part of May 1818 by RECAMIER,[4] and since recommended by JOBERT, LIS-

1 Henle: Anatomie des Menschen. Band II., p. 482.

2 J. V. Gairal: Des Descentes de Matrice; Traitment des Maladies du Col par les Liquides. Paris, 1872, p. 107.

3 Hygiea: Svenska Lakaresallskapets Forhandlingar. 1875, p. 157.

4 J. V. Gairal, op. cit., loc. cit.

FRANC, VELPEAU,[1] E. MARTIN[2] and others. As sufficient experience is already collected for us to be able to form for ourselves a reliable opinion as to the different values of these caustics, it is not devoid of interest to hear the different opinions which writers have expressed concerning these agents; and on this point I will premise in brief, that all who in daily practice make use of caustics, have become chargeable with producing stenosis of the cervix.

In regard to sulphate of zinc, SKOELDBERG[3] says: "I found that the effect of the sulphate of zinc (in crayons, according to BRAXTON HICKS' method) was very powerful, and I also saw two cases in which a cicatricial adhesion of the os externum had taken place after its employment; I therefore determined to find some means for mitigating this too powerfully caustic influence." On this application (zinc-alum crayons) FRANKLIN NYROP[4] makes, among others, the following remark: "Sometimes also very considerable contractions of the cervical canal may arise therefrom. . . . With Prof. HOWITZ I have seen in a patient, who was treated by himself, so considerable a stricture, that only a fine sound could pass the canal, for which it was necessary to perform bilateral dilatation." On the nitrate of silver pencil, E. MARTIN expresses the following opinion: "Contracting exudations after inflammations of the inner margin of the lips of the uterus, with or without ulceration, under certain circumstances, undoubtedly produce strictures of the internal or external os uteri and cervical canal, particularly if the inflammation has attacked the entire circumference of the mouth of the womb. A similar contraction of the os uteri is induced, as my own experience and the practice of others have taught me, by repeated cauterizations with nitrate of silver in substance: while scarcely in a single

---

1 Henri Despeyroux: Etude sur les Ulcerations du Col de la Matrice. Paris, 1872, p. 112.

2 E. Martin, op. cit., loc. cit.

3 Nordiskt Medicinskt Arkiv. Band I., N:r 9, 1869, p. 6.

4 Bibliothek for Læger. Band IV, Heft 2, 1874: Den Intrauterine Behandling, p. 300.

instance have I observed this result after numerous cauterizations with acid solution of nitrate of mercury."

Let us now hear the experience of BERNUTZ and GOUPIL[1] in regard to the agent last mentioned. These very eminent authors express themselves on this point in the following manner: "A case of 'cicatrisation' laterale gauche du col,' which we observed in 1848 jointly with PIEDAGNEL, had been produced by cauterizations with liquor nitratis hydrargyri, and gave rise to very severe dysmenorrhœa, but did not prevent a complete discharge of the menstrual blood." Another case was that of "a young lady, who was the subject of metritis with granular ulcerations of the neck, extending as far as the interior of the canal. The ulcerations were cauterized with liquor nitratis hydrargyri and Filhos' caustic (solid Vienna paste). The result of treatment was occlusion of the external os, which was relieved by vaginal hysterotomy." Not without reason, therefore, BERNUTZ and GOUPIL warn against the employment, without sufficient caution, of a therapeutic agent, of which so deplorable an abuse is made.

I have mentioned above what an excellent application tannin crayons are in the lighter forms of uterine catarrh. But in the severer cases, where the ulcerations are more extensive, the papillæ having become hypertrophied or neoplasms of the same structures having taken place, a more powerful agent undoubtedly becomes necessary, and this is the sulphate of copper, which for a century past has been extensively employed without meeting with discredit. On this STELLWAG VON CARION[2] expresses the following favorable opinion: "When the mucous membrane is very greatly swollen and relaxed, the catarrhal secretion being quite abundant—and it is, consequently, more a question of a strongly astringent influence than of a powerfully caustic effect—sulphate of copper in crystals is unconditionally the best agent." I have had no little experience with this remedy in dilute form (1:5), which I

1 Clinique Medicale sur les Maladies des Femmes. Par G. Bernutz et E. Goupil. Tome I; Paris, 1860.

2 Dr. Karl Stellwag von Carion: Lehrbuch der Praktischen Augenheilkunde. Wien, 1864, p. 402.

daily employ in the severer cases of uterine catarrh for painting with PLAYFAIR's sound[1] over the entire interior of the uterus, and can testify that it is especially efficacious without being the cause of any inconvenience. It does not erode the mucous membrane of the cervical canal like crayons of nitrate of silver, sulphate of zinc, or the zinc-alum crayons; but I have applied it without producing this injurious effect upon the follicular structures, in which catarrh has its principal seat.

I have remarked above, that we must make an accurate distinction between those causes which are the most commonly occurring, or at least those which in the majority of cases or with great certainty produce the strictures in question, and those which only very rarely make their influence felt. Now, belonging to the former class and standing in the foremost rank, according to my opinion, is the abuse of solid caustics, producing deep solutions of continuity when employed in the treatment of diseased conditions within the cervical canal; to the latter, on the other hand, belong certain pathological conditions which arise independently of treatment. That is to say, it is altogether impossible for us to agree with SCHRŒDER's above-mentioned theory, that traumatism resulting from parturition, and the puerperal inflammations, are the foremost among the producing causes. In opposition to the former of these is the consideration, that notwithstanding the fact that the lacerations ordinarily occurring in the cervix are all healed with a cicatrix, yet the calibre of the cervical canal—thanks to the enormous dilatation of the cervix during parturition—I may say, without exception after child-birth, shows a dilatation which is most considerable at the external os, or just at the point where the lacerations have been deepest and most numerous. And, that we should as little adjudge to puerperal inflammations any prominent influence in the production of stenosis, is indicated by the fact, that as often as we meet with these inflammations in the uterus and its annexa, so seldom can we regard stenosis of the cervical canal as the

1 [See Ziemssen's Cyclopædia (Am. Ed.) Vol. X., p. 136—also Brit. Med. Jour., Dec. 11, 1869, and Lancet, 1870, II., July 1.]—TRANSLATOR.

sequelæ of these. The same is true with regard to blennorrhœa of the cervix as a cause of strictures. Blennorrhæa occurs very commonly in prostitutes, but stenosis of the cervix in this class of women is, as above stated, extremely rare or else little noticed. SCHRŒDER's supposition that strictures are produced in consequence of rupture of the ovula Nabothi, the granulating walls becoming adherent to each other—it is quite certain we very rarely indeed have the opportunity of observing, if ever at all.

Among such causes as very rarely exert their influence, those perhaps should be reckoned which are mentioned by Martin, viz: deep spontaneous ulcerative lesions occurring during pregnancy; likewise diphtheritic formations, and vaginal injections with caustic liquids; besides, in the non-gravid, contracting exudations after inflammations of the inner margin of the lips of the uterus, with or without ulceration.

CLASSIFICATION.—On the ground of the experience acquired, during the observations which I have had the opportunity of making, in regard to strictures, of the cervical canal, in the proper sense of the term, these perhaps may be divided into *obliterating*, *cicatricial* and *callous*. The obliterating may, in harmony with the commonly received method of speaking, be subdivided into *totally obliterating* or *adhesive in the proper signification*, and *impermeable in the surgical sense*, which latter are either *adhesive* or *ethmoid*. Of the callous I have observed three different kinds, viz: *circular*, *semi-circular* and *diffused callous*.

### I.—OBLITERATING.

A. *Totally Obliterating or Adhesive in the Proper Sense.*—These are recognized when the walls of either one or both of the orifices of the cervical canal, or the walls of the canal itself in its entire extent, or in any portion of it, are completely adherent to each other. The totally obliterating strictures are well known to the pathological anatomists. Thus FŒRSTER,[1] for instance, remarks: "From adhesions after inflammation, atresia may occur in partic-

1 Lehrbuch der Pathologischen Anatomie. Von Dr. August Fœrster: Sechste Auflage. Jena, 1862, p. 481.

ular portions, or obliteration of the entire cervix." ROKITANSKY[1] has observed, that "obliteration of the os externum alone is rarely the case, when cicatricial formations occur after ulcerative loss of substance." KLOB[2] says: " . . . obliterations of the cervix uteri occur especially in the ostia," and "the granulations[3] which are developed from the ulcerated surfaces likewise often lead to atresia;" also further on:[4] "incontestably it comes in many cases from adhesion through the epithelial structures, succeeded by a firmer closing up of the adherent tissues, but which often, too, limits itself to merely the external extremity of the canal."

In consequence of the fusiform cervix in virgin uteri ("virgines quoad uterum") they occur principally in the ostia—according to KLOB,[5] oftener in the internal than in the external; according to COURTY,[6] on the contrary, they generally affect the lower portion of the canal. My own experience is too limited to decide this disputed question. As an example of such strictures and their origin, I present the following case:

*Case I.*—K. J—n, a seamstress, 30 years old, from Stockholm.

As a child she was always very delicate, was very frequently sick and kept her bed. The menses appeared for the first time when she was sixteen years old, without pain, and continued for three or four days. Afterwards they were absent for nearly a whole year, when she was very weak and delicate. When they subsequently returned they continued each time from three to four days, but were always preceded by and accompanied with severe sufferings, and were also followed by leucorrhœa. The intervals between the periods comprised usually eight, nine or ten weeks. In the year 1872 they were again absent

1 Lehrbuch der Pathologischen Anatomie. Von Carl Rokitansky, Band III., Wien, 1861, p. 466.

2 Pathologische Anatomie der Weiblichen Sexualorgane. Von Prof. Dr. Jul. M. Klob. Wien, 1864, p. 109.

3 Klob, op. cit., p. 111.

4 Klob, op. cit., p. 113.

5 Klob, op. cit., p. 109.

6 Traite Pratique des Maladies de l'Uterus, des Ovaires et des Trompes. Par A. Courty: Deuxieme Edition. Paris, 1872, p. 398.

three or four months, and she therefore consulted a physician, who succeeded, by the administration of iron, in restoring the menses to their proper time. At the same time, for her leucorrhœa, applications of zinc-alum crayons were employed in the cervical canal. During the progress of this treatment she had at one time a hemorrhage contining for four weeks. She came under my care on the 27th of January 1874, for chlorosis, gastric catarrh and ulcerations of the cervix. So far as we can be considered authorized in drawing any conclusion from the patient's social condition, morals, habits, etc., we must here rely upon her statement that she has never had intercourse, and far less has she ever been affected with blennorrhœa. Under the use of Karlsbad water and iron, the gastric catarrh improved and her strength increased; but at the same time it happened that under the application of nitrate of silver a contraction of the os externum took place; and in this way, besides, the raw surfaces coming together became completely united with each other, so that total obliteration of the external os occurred. I expected now that the dysmenorrhœal pains during the next following periods would be augmented to their greatest severity, or that symptoms of retention, or of retrouterine hæmatocele, might appear; but, strangely enough, all these troublesome consequences failed to occur. She was operated on by the introduction of a trocar into the cervix and bilateral incision with SIMS' knife. She afterwards had her menses regularly, the last were shortly after July 1875, but since this they have failed to appear. The external os is again nearly occluded, and I have now advised her to submit to SIMON's conical-plastic ("kagelmantelformiga") excision of the cervix.[1]

*B. Impermeable Strictures, in the Surgical Sense.*—These

---

1 [Literally, "conical-mantleform."

*See* Ziemssen's Cyclopædia of the Practice of Medicine. Vol. X. Diseases of the Female Sexual Organs. By Carl Schrœder. (American Edition), pp. 79–80. Amputation of the infravaginal portion.

*See*, also, notice of Simon's "Wedge-flapped Excision of the Cervix Uteri and its Applications;"—review of Dr. M. Marckwald's paper (Arch. fur Gyn., Band VIII., Heft 1.)—American Journal of Obstetrics and Diseases of Women and Children, Vol. VIII, No. 3, pp. 564–5. *Also*, Ziemssen's Cyclopædia. (Am. Ed.) Vol. X., pp. 90–93.]—TRANSLATOR.

strictures are characterized by the fact that the walls of the cervical canal to a greater or less extent, or of one of its orifices, are incompletely united with each other, so that the menstrual blood may ooze through them very well, but it is impossible to pass even the finest sound. They are subdivided into (a) the adhesive, and (b) the ethmoid.

a. *Adhesive Impermeable Strictures.*—In these the walls of the cervical canal or those of one of its orifices are firmly united with each other, yet not so completely as to prevent the menstrual blood and uterine secretions from trickling away, through one or more capillary openings, which still do not allow the finest sound to pass. The following may be related as an example:

*Case II.*—A. S., a servant girl, aged 37, from Stockholm. She became regular for the first time between her eighteenth and nineteenth years. The bleeding continued only one day, and was neither preceded by nor accompanied with pains. The menses afterwards returned regularly after intervals of four weeks, yet always scantily; at twenty years of age she became weak and easily fatigued, and experienced also other symptoms of chlorosis; at twenty-six years of age the menses disappeared and were absent for a year and a half, but returned after the use of medicines which were administered for the chlorosis. Afterwards she had her menses regularly until three years ago, when during a journey from Hasthœlmen to Linkœping they failed entirely to appear and did not return until the following spring, after which they returned at intervals—now of three, now of five weeks—and continued for four days. For lassitude and aching limbs, besides a thick yellowish discharge with which she has been troubled for a long time back, (there is no question that she has been affected with blennorrhœa), she consulted several physicians, with the result that her general condition was improved and the discharge diminished, and became clear and colorless. In October 1874, she began to undergo local treatment with zinc-alum crayons, from which she experienced severe pains in the loins, and the discharge now became yellowish, and purulent and "cheesy clots" (caustic eschars) came away. With the pain in the lumbar region she had already long before been troubled, every other time and one day before the catamenia should appear, which pain ceased with the beginning of the bleeding. Concerning the abundance of the catamenia in the winter and spring of 1875, she made the observation that as long as she remained very quiet, only a little blood came away; whereas, when she exerted herself much the bleeding became very copious. In the beginning of June 1875 she was seized again with the most violent pains in the lumbar region before the period, which continued for four days, yet extremely scantily, so that only a few stains appeared upon her linen; at the same time she was troubled with a smarting in micturition.

*Status præsens*, July 10th, 1875.—The patient is of small stature, full habit, but lax in flesh, she looks like an extreme sufferer, anxious and melancholy. She states that during the period, which terminated two days ago, the sufferings were worse than ever.

With bimanual examination the uterus is found to have taken an anteflexed position and not to be enlarged. Neither at the sides, above nor behind the uterus, can any resistance be felt. The vaginal walls are wide and relaxed. The vaginal portion is conical, yet but slightly abnormal. Through the anterior vaginal roof the uterus may be palpated, the body of which is bent upon the cervix at nearly a right angle and deviates also to the left (antero-lateral flexion). The os uteri presents the appearance of a fine transverse cleft nearly a centimetre long, the extremities of which are formed by two small holes, the size of a pin's head, in the mucous membrane. In the attempt to introduce the uterine sound it is found that the anterior and posterior lips of the os uteri are almost completely united with each other through a strong membrane, which in appearance and consistence exhibits the same qualities as the rest of the vaginal portion. It does not yield in the least when pressed upon with the sound. The small openings just mentioned, the size of a pin's head, at the extremities of the rima, do not at all permit the finest elastic sound to pass, but it is through these that the menstrual blood and uterine secretions ooze away, which was observed during examinations made twice a week up to the 9th of August, 1875; at which date an incision of the adherent uterine lips was made, and extended in the cleft between the pin-head openings; after which the strong, but not cartilaginously hard, connecting membrane—which united the anterior and posterior cervical walls—was divided with a fistula knife. The uterus was then sounded twice a week.

October 15th, 1875.—The woman's menstruation now takes place without any pain, but the subacute gastric catarrh, notwithstanding an exact diet, etc., manifests itself as continuing at the same point. AVELING's sound can with ease be introduced into the uterus.

b. *Ethmoid Strictures.*—In these the walls of the cervical canal, to their whole extent or only partly, are united with each other through extremely strong and hard fibres of connective tissue, which only with difficulty are cut through, whereupon they crackle under the knife and do not give rise to any bleeding worth mentioning, as they are very poor in blood vessels, and for this reason also they present a pinkish or grayish appearance. Retiform strictures are described by KLOB[1], who says: "the atresias extending over the greater spaces affect most often the cervical canal. The structure, which in such cases produces the occlusion, is a soft, sometimes vascular, connective tissue, which—under the form of filaments and lamellæ—stretches from one wall to the other; and often the valvular spaces between these lamellæ, being filled with serum, are closed up; for which reason it can only with difficulty be determined, how far they are the remains of former follicles or—which is more probable—give evidence of an incomplete obliteration of the cervical canal. In most cases, we can with a steel sound, without the employment of force, separate these bands of connective tissue from each other," etc.

Besides the above enumerated diagnostic signs and general characteristics, the ethmoid strictures of my own observation differ from those, which KLOB has described,

1 Klob, op. cit., p. 111.

in the fact also that they do not give way even before the strongest pressure with the point of the sound.

In the five cases which I have observed, the patients had been treated beforehand for ulcerative cervical catarrh by the introduction of zinc-alum crayons into the cervical canal. The origin of these can be explained by the exfoliation of the mucous membrane of the cervical canal as caustic eschars, and the healing of the consequent solutions of continuity through the formation of granulations. These neoplastic granulations from the anterior and posterior walls of the cervical canal have directly coalesced and given rise to the filaments of connective tissue of a tendinous firmness. COURTY[2] also has described these strictures, but GALLARD[3] denies their existence, and expresses himself as unaware that such strictures have ever been observed or described by any author. I term them ethmoid or sieve-like, because the flow of menstrual blood, and of other uterine secretions, traverses them without much interruption, as if through a sieve or filter, under the influence of the rythmical contractions of the uterus. In the five cases observed by me the patients have been sterile. Permit me to relate the following three cases.

*Case III.*—J. G., seamstress, aged 28, from Stockholm. After overstraining herself, she had a hemorrhage from the genitalia for the first time when sixteen years old. She became faint and terribly ill with the pain over the back and abdomen. The bleeding continued for two days. She became regular at seventeen years of age. Menstruation continued three or four days and took place entirely without pain. It since returned regularly every four weeks, has usually lasted three to four days, at a later time five to six. Before the ingress of menstruation she has not generally had any pain until latterly. She has been troubled with chlorosis for a very long time, and with a discharge ever since the establishment of the catamenia. The chlorosis has been on a steady increase, with pain in the back and abdomen, and also great weakness.

For the past six years she has suffered with sometimes

2 A. Courty, op. cit., p. 682.

3 T. Gallard: Lecons Cliniques sur les Maladies des Femmes. Paris, 1873, p. 188.

violent, sometimes less severe "grinding" pains in the lumbar spine and cramps about the stomach, which symptoms sometimes, but not always, increased shortly before the catamenia, but always became most severe during the progress of menstruation, and especially towards its close. For three years past she has been at the Seraphine Lazaretto for gastric inflammation. She was afterwards attended by another physician for ulcer of the uterus and inflammation of the ovaria. The local treatment consisted in the introduction of zinc-alum crayons and tampons. The cramps over the lumbar spine, hips and abdomen, during the periods themselves, with which she had already suffered before, at the last time all at once became augmented and manifested themselves as "grinding pains," during which the discharge of blood was observed to be more abundant, whereupon the pains abated, again to advance by degree to the paroxysm.

In the patient's statement, that she has never had intercourse and still less been affected with blennorrhœa, I believe that I can place full confidence.

*Status præsens*, September 9th, 1875.—The patient is tall and thin, with a grayish-yellow dark complexion, and seems to be an extreme sufferer. She complains of weakness of the limbs, acidity of the stomach, and headache; besides which she is annoyed with an uneasy feeling in the stomach.

The examination indicates chlorosis, gastric and intestinal catarrh, and also leucorrhœa.

The uterus is not enlarged, but anteflexed. After the sound is passed halfway into the cervical canal, it is stopped by an obstacle, which—after dilatation of the lower portion of the canal and holding the lips apart—is found to depend upon an adhesion of the upper portion of the canal, through short but very firm and hard bands of connective tissue. These were completely divided by incisions with a fistula knife, after which the patient's uterus was sounded twice a week. Menstruation afterwards occurred without pain.

*Case IV.*—Mrs. A. M. F., aged 33, from Stockholm. Married since May 3d, 1874. Menses appeared for the first time in her fourteenth year; but she cannot remember how many days the menstruation continued, yet she recollects that it occurred without pain. Menses since returned regularly after the expiration of four weeks, and the bleeding lasted usually eight days, which she attributes to her having been delicate and having also been obliged to work "dreadfully" hard. At twenty-one years of age, she became pregnant and had a tolerably easy labor. Her lying in seems to have occurred normally, at least she left her bed on the second day and attended to her usual duties, but she was one time troubled all along with a thick, yellow discharge of a disagreeable odor. Five years afterwards she had a miscarriage at the fourth month and then lay in bed some time with fever. Thereafter, she believes as the consequence of over-exertion, she began to be troubled with pains in the abdomen, not only before menstruation, but also after its cessation.

Her present attack she dates from July, 1872. She had then had her menses for four days, during which—in the same way as before, and afterwards, so many times—she must have over-exerted herself very considerably by carrying heavy burdens. She then had a violent pain in the left groin and abdomen directly above the vulva, but this attack was relieved after the administration of a purgative. Somewhat more than a year ago, as she was very much troubled with abdominal pain and a flux, she consulted a physician, who pronounced her disease to be "an ulcer of the uterus;" for which, during a further period, treatment with zinc-alum crayons and tampons was employed. She became free from the pain, and the whole summer of 1874 was without suffering, and comparatively free from the flux. She positively denies that she has had any discharge, that can be construed as blennorrhœa.

*Status præsens*, June 7th, 1875.—The patient is rather tall and thin, but has a very bright, red-cheeked appearance. The expression of her countenance, nevertheless, gives indication of a certain oppression, mingled with anxiety. She is subject to chlorosis and also gastro-enteric catarrh.

With bimanual examination the uterus is found not to be enlarged, and occupies its normal position in the pelvis. The vaginal portion is of moderate size, perhaps somewhat flattened from before backwards, but neither doughy nor livid; on the contrary, it is felt to be firm and hard, and also has a bright, rose-red color. The os uteri forms a transverse fissure. On separating the lips of the os from each other, the walls of the cervical canal are perceived to have been united with each other through thick, hard and strong adhesive bands, which made it impossible for the point of the sound to enter even a line into the cervical canal. In cutting through the connecting fibres just mentioned with a fistula knife, so that the natural width and form of the cervical canal was restored, they crackled under the knife at every point, and were observed to fill up the whole length of the canal as far as the os internum.

The woman has since this been sounded regularly twice a week. The cervical canal now permits the sound to pass with ease, and her menstruation takes place entirely without pain.

*Case V.*—Mrs. C. A., aged 38 years, from Stockholm, married since twelve years ago. As a child she was not very robust, and was troubled with chlorosis before the first advent of the catamenia. This occurred when she was fourteen years old, the bleeding continued two or three days and was preceded by pains in the back. Menses since returned regularly after four weeks' intervals, lasted generally five to six days and were preceded usually by pains in the back, which ceased after menstruation came on. She has borne three children. Her two first labors were very easy. The last, which took place eight years ago, was very difficult. Previously, during pregnancy, she had been much troubled with constipation, and the most violent labor pains continued for two days. She had no important hemorrhage after the last parturition, but the placenta was manually delivered. Her present disorder (retroflexio uteri) she dates from the last delivery, particularly the severe pains in the upper portion of the hypogastrium, which were experienced as "a constant aching, which was indeed never relieved."

These pains in the lumbar spine and right groin have gradually become altogether more violent before the ingress of menstruation, and she has between the periods been much troubled with a discharge and general debility, especially a weakness in the legs and back. She has for several years past been treated by various physicians locally, with applications of zinc-alum crayons. The discharge after this was improved, but she had pains in the right inguinal region, and finally became so weak that she "staggered." During the close of 1874 and the year 1875 up to the month of August, the pains have been, before and during menstruation, still more augmented, whereas the condition has been the opposite in regard to the bleeding, which became more scanty, so that the last time scarcely any blood came away. The condition has for the most part been such that when the aching in the right groin and back had lasted some time, a little blood appeared, after which the bleeding stopped for a half or a whole day, and a "twinge" was felt throughout the intermission.

*Status præsens*, July 15th, 1875.—The patient is small in stature, but quite disproporately fat and gross. She is very pale, but does not present any further appearance of sickness, though she is quite anemic.

The uterus is found to be retroflexed, and the entire cervical canal grown together through firm, hard and stiff connecting fibres, which were cut through with a fistula knife, whereupon they crackled just as when we cut through the tendo Achillis. Afterwards the uterine cavity, which was not inconsiderably dilated, was sounded twice a week, and the patient was relieved of her retroflexion through the employment for some time of AMANN's stem. She was in good health, until on the 5th of October 1875 she returned, suffering with intermittent malarial fever, but states that her pelvic organs are in sound condition.

II.—CICATRICIAL STRICTURES.

These, as I have had the opportunity of observing—and all of which occurred in women who had not borne children—have had their seat in the external os. They are recognized when the os externum is unusually small, a punctiform opening, surrounded by a great number of cicatricial furrows, of an ivory-whiteness, more or less deep, so that the external os itself resembles the central depression of a radiating cicatrix. It is impossible to introduce the common uterine sound through the stricture, which permits only a very fine elastic sound to pass. When the lips of the os are completely united with each other, these strictures become obliterating.

In consequence of the fusiform cervical canal in virgins ("virgines quoad uterum"), these have their seat principally in one or both ostia; according to KLOB, oftener in the internal than in the external os. How much they owe their origin to the original disease (ulceration), and how much should be attributed to the employment of powerful caustics, it is in a given case almost impossible to determine. It is certain that from a mild erosion a superficial ulceration may arise through a single application of nitrate of silver, which is usually healed without contraction, if we only desist in time from the use of the strong cautery. On the contrary it may happen, especially under repeated applications of powerful caustics, that from the superficial, a deep ulceration proceeds, which cannot be healed without producing a diminution in the calibre of the canal.

*Case VI.*—A. J., a midwife, aged 27, from Stockholm. She was healthy as a child. In her thirteenth year she began to be troubled with chlorosis. She had her menses for the first time when fourteen years old. They were preceded by and accompanied with severe pains, and the bleeding was very copious, but returned irregularly after intervals of eight, fourteen to twenty-four days. At no time did the interval between two periods constitute twenty-eight days. This state continued until she was twenty years old, when she was attacked with violent hemorrhages from the lungs. Menstruation then became more regular, returned generally after four weeks' interval, and continued three, at most four days, but the bleeding was very slight. In her twenty-fourth year the menses were absent during three months' time, without her experiencing thereby any suffering. At twenty-five years of age she was troubled to such a degree with pains in the abdomen, and particularly with an aching of the lumbar spine and in the inguinal regions before and during menstruation, that she was obliged to call in a physician. The local treatment consisted in repeated introductions of zinc-alum crayons.

*Status præsens*, March 10th, 1875.—The patient is of short stature, blonde, and quite fleshy. She is very pale, but otherwise has no appearance of sickness. Loud anemic sounds are heard over the vessels of the neck, and she complains much of weariness in the limbs, dizziness, and other symptoms of chlorosis, besides which she is troubled with chronic gastric catarrh.

With bimanual examination the uterus is found to be retroverted, but no worse than that its fundus is situated quite below the promontory. The mucous membrane of the vagina and of the vaginal portion, is intensely red. The vaginal portion is nearly four centimetres long and has a typical conical form; the external os is directed forward towards the vulva, and consists of an unusually small, punctiform opening, which is surrounded by a great many cicatricial furrows, so that the external os itself resembles the central cavity of a radiating cicatrix. A fine elastic sound (French scale, No. 5), only passes with difficulty into the uterus, which is not enlarged.

When the attempt to dilate the os uteri and cervical canal, by the introduction of larger elastic sounds, was unsuccessful, an operation was performed, on the 7th of July, at the patient's home, without chloroform. She was placed in SIMS' position on the right side, and SIMS' speculum and a retractor were then introduced. The uterus was fixed by means of a SIMS' hook; then by the employment of KUCHENMEISTER's scissors, bilateral incision of the lower third of the vaginal portion was performed; after which SAVAGE's double-bladed hysterotome divided the remaining portion of the stricture as far as up to the neighborhood of the internal os. Some arterial bleeding was checked immediately by the injection of ice-cold water, after which the wound made in the operation was, by means of a PLAYFAIR's sound, penciled with chloride of iron, and a tent of hæmostatic cotton introduced into the cervical canal. The vagina was carefully packed with boracic acid tampons. The patient was carried to her bed. No unfavorable reaction occurred after the operation.

The dressing was changed daily, and on the 12th of July, and from that date, the patient was sounded twice a week with an elastic bougie No. 20, which was easily introduced up to the fundus uteri. She experienced a remarkable improvement in her sufferings after the operation, but still has the retroversion, as she cannot undergo the stem treatment ("stiftbehandling"). She is troubled very constantly with pains in the lumbar spine and groins; besides which, her chlorosis and gastric catarrh continue, though improved.

October 19th.—An elastic bougie No. 18 passes easily to the fundus.

III.—CALLOUS STRICTURES.

These are recognized when the calibre of the cervical canal, in its whole extent or partially, is diminished through a prominent, cartilaginously hard, new formation of the connective tissue.[1] These strictures are perfectly analagous to those which occur in the male urethra, and on which VON PITHA[2] says: "Of all kinds of strictures the so-called callous are the most often observed; of the inveterate gonorrhœal strictures nearly all belong to this form." In accordance with what has been remarked above, it is still in dispute as to how much the origin of these strictures should be attributed to the disease (gonorrhœa), and how much to the treatment with strong caustics. Two of my patients (cases VII and VIII) had undoubtedly had blennorrhœa; but both had also been treated with repeated applications of zinc-alum crayons to the cervical canal. I do not hesitate to maintain that if these patients had been treated first antiphlogistically and afterwards hydro-

1 Heteroplasm of the submucous areolar.—TRANS.

2 Von Pitha: Handbuch der Speciellen Pathologie und Therapie. Band VI., Abtheilung II. Krankheiten der Mænnlichen Genitalien und der Harnblase, p. 184.

therapeutically, or with local astringents, the strictures would not have occurred.

A. *Circular Callous Strictures.*—Here the hard cartilaginous tissue, round about the canal, encroaches upon its calibre, extending to a greater or less length. In the former case the ring is like an elevated ridge, in the latter it has sometimes a sharp, projecting edge.

As an example of circular callous strictures of the latter class, allow me to mention

*Case VII.*—A. C., a seamstress, aged 29, from Stockholm. She was very frail and feeble as a child, and has also been troubled with chlorosis and disordered stomach as far back as she can remember. She became regular at fifteen years of age, but cannot state how far the bleeding was preceded by, or accompanied with pain, nor how long it continued. Yet she knows with certainty that after a difficult labor which she had six years ago, it was always very irregular and was preceded and attended by severe pains in the lumbar spine and groins. She does not deny that she has been affected with blennorrhœa, for which she was treated locally by the application of solid caustics (zinc-alum crayons). For a long time past she has been complaining of continually increasing debility and undefined pains in the abdomen.

*Status præsens*, May 29th, 1875.—The patient is of small growth, very thin and delicate, and has also the appearance of being consumptive. She complains of constant pains between the breasts, on the right side above the crest of the ilium, in the inguinal regions and lumbar spine. She is very anemic, and besides this is afflicted with gastro-enteric catarrh and retroversio uteri. The anterior lip of the uterus is fixed to the anterior roof through a short, sharp bridle, half an inch broad, which in the attempt to replace the retroverted uterus resisted considerably. The attempt at reposition gave the patient acute sufferings. The external os is a wide transverse cleft. An attempt to introduce the sound was unsuccessful, which was due to the fact that a centimetre from the external orifice, there was discovered in the cervical canal an annular callous stricture, which only with the greatest difficulty allowed a fine elastic sound (French scale No. 5) to pass into the uterus, which is of the normal size.

The stricture was now dilated by degrees with elastic sounds until No. 9 could be introduced, it was then incised bilaterally on a director, previously introduced, with a sharp fistula knife; after which a larger sound (No. 18) could be introduced. The patient was afterwards sounded twice a week with the ordinary uterine sound. She was for a short time subjected to the stem treatment after AMANN's method, but experienced merely an inconsiderable alleviation on account of the resistance of the above-mentioned bridle, which I did not venture to cut through on the examination table in my consultation room, as Barnes has cautioned against it. Besides, the woman had, like the most of my patients, no time when she could desist from her fatiguing work, so that any additional operation at her home could not be considered.

She settled afterwards in a damp locality, on a low marsh; and was attacked with intermittent fever and a violent metrorrhagia, which did not yield until after the use of quinine internally and painting the interior of the uterus with fuming nitric acid. She now got up some time subsequently and attended to her vocation, but was again taken sick with perityphlitis, of which she died in August, 1875.

At the *post mortem* examination the mucous membrane of the body of the uterus and of the cervical canal was found to be very pale, of a glassy clearness, and glistening. Nowhere could any concentric atrophy of the uterine cavity, as a consequence of the impenciling with fuming nitric acid, be detected. In the cervical canal the rugæ of the mucous membrane had disappeared, but the canal had its normal form and proper calibre. Of the stricture no trace was seen. Surrounding the cæcum there was quite a large abscess, which proceeded from the appendix vermiformis, but in the true pelvis the peritoneum was pale and free from pus.

B. *Semicircular Callous Strictures.—Case VIII.*—Mrs. N. N., aged 36 years, from Stockholm. Married since twelve years ago, she has borne one child still living, which is eleven years old, and she has also had two miscarriages. She became regular for the first time when seventeen years old. Menses appeared without pain, but she cannot remember how long the bleeding continued. Menstruation since returned regularly after four weeks' intervals, has continued three to four days and without pain. For about three years past she began to experience a difficulty in passing water, consisting in quick urgent cramps in the evacuation of the urine, besides an indescribable smarting after urination, and a thick yellowish discharge (blennorrhœa), which she believes she had possibly contracted from her husband. She then began to consult a physician and was treated locally with applications of zinc-alum crayons to the cervix. Latterly she has, though never previously troubled with any pain before or during menstruation, sometimes felt an aching in the lumbar spine, also stinging and lancinating pains in the right groin; but the menstrual blood came away as formerly, without a pang or anything different from before.

The patient was treated during the summer of 1874 and the winter of 1875 continuously with applications of zinc-alum crayons in the cervix.

*Status præsens*, July 10th, 1875.—The patient is quite tall, of large frame, with a very good supply of flesh. Her face is very pale; sorrow and physical suffering have left their impress there. She complains of constant distress in the abdomen, where she feels a heaviness and downward pressure. She is very anemic, her appetite is delicate and evacuations sluggish.

With bimanual examination the corpus uteri is found to be absent from its proper place in the pelvis and somewhat lower than common, but not enlarged. Somewhat more than an inch within the vulva the anterior lip of the uterus is reached, considerably swollen, having a hard, elastic feeling and a bright rose-red color. The os uteri forms a long transverse slit. Quite within the external os there is discovered—proceeding from the left side of the cervical canal and encroaching far in upon the canal—a ridge (semicircular, callous stricture), which occluded nearly the entire lumen of the canal, so that only along the right wall enough space remains for a fine elastic sound (French scale No. 7) to pass with great difficulty into the uterus, the cavity of which is of normal length.

The patient left for her home (Gotland) and was again taken sick there with retro-uterine hæmatocele. After her return to the capital (in August 1875) she was so far restored to health that I believed I could begin to dilate the stricture of the cervix, which was done by introducing gradually larger elastic sounds (from No. 6 to No. 9). After the stricture became so much dilated, a director could be introduced, on the groove of which the stricture was cut through with a fistula knife. No bleeding. A tampon was applied to the cervix.

The patient was afterwards sounded regularly twice a week.

March 2d, 1876.—The patient has a retro-uterine hæmatocele, which extends up to the umbilcus.

*Case IX.*—Mrs. S. G., aged 31 years, from Stockholm. As a child she was quite strong, stout and fat. She became regular in her eighteenth year, without pain. The menses continued the first time for three days. They then returned regularly after four weeks and continued four days. At twenty years of age she began to be troubled with chlorosis and leucorrhœa. At the same age she was married. She has borne three children. During the last confinement she had pains in the stomach, but was relieved by the use of morphine and poultices. Yet she was troubled from that time with "a bad, distressing, yellow discharge," as if from a sore in the uterus, for which she was treated by a physician with repeated introductions of zinc-alum crayons; after which she noticed long, thick pieces of slimy membrane coming away, and copious bleeding following. Since this she has suffered from continually increasing pains before and during the menses, which became very irregular, at one time lasting two to three, at another time eight to ten days, during which the bleeding had an intermission of one or two days. Between the menses the abdomen has been very tender. She has not had blennorrhœa.

*Status præsens*, July 20th, 1875.—The patient is of small stature, has a pretty good supply of flesh, and does not seem much sick. She is troubled, notwithstanding, with gastro-enteric catarrh and also fluor albus. The cervical canal allows only along its right wall a very fine elastic sound to pass, because immediately within the external os, from its left wall arises a ridge-like, semicircular cartilaginous growth. This having been cut through with a fistula knife, the patient was sounded twice a week.

March 1st, 1876.—The common uterine sound enters with ease to the fundus, but the patient continues to complain of her sterility.

C. *Diffused Callous Strictures.*—As the name implies, in these strictures the hard, cartilaginous structure has not assumed any determinate form, but spreads itself—to a greater or less degree constricting the calibre of the canal—unequally over different portions of its walls.

As an example of these, permit me to introduce the following:

*Case X.*—Mrs. E. M. H., aged 42 years, from Skonen. She reports that during her earlier childhood she in general enjoyed good health; but after she had attained her twelfth year, she began to be troubled, more and more every year, with chlorosis, which she ascribes to overexertion and meagre fare. Her menses appeared the first time when she was sixteen years old. The interval between the periods has usually constituted twenty-six days, and the bleeding has in general not gone on for more than two days, but always preceded by and attended with intolerable sufferings in the lumbar spine. Before, and particularly directly after, menstruation she has been much troubled with "a severe discharge," which considerably diminished her strength.

She has, in her twenty-three years' marriage, given birth to five children, of whom only two are living. She has had two miscarriages. The last conception which terminated in this way took place eight years ago.

Ever since the first parturition the above-mentioned pains in the lumbar spine during menstruation have been less severe than before. She was treated for several years with Hodge's pessary and the application of solid caustics

(zinc-alum crayons) to the cervical canal, for ulcerative cervical catarrh. She has never had blennorrhœa.

*Status præsens*, September 10th, 1875.—The patient is of medium height, thin and pale (anemic). She is troubled with symptoms of a long continued gastro-intestinal catarrh, and has suffered besides for a long time past with retroflexio uteri.

The os uteri, which was formerly a transverse fissure, is now a nearly round opening, surrounded by callous grooves, and allows a common uterine sound neatly and tightly to pass. The cervical canal itself is likewise considerably narrower than normal, and its walls are felt with the beak of the sound to be very uneven and unusually hard and cartilaginous. The orificium internum is passed with comparative ease by the point of the sound.

As the stricture is not any worse than will allow the introduction of an Amann's stem No. 5, with which the patient feels easy and content, it is considered that no operative interference is required.

DIAGNOSIS.

*The diagnosis* of strictures in general is in most cases not subject to any uncertainties. In those strictures situated at the orificium externum and in the lower portion of the cervical canal, an inspection is sufficient: in those found higher up in the canal and at the isthmus, our attention is attracted by the obstacle thereby presented to the passage of the ordinary uterine sound. We pass on then to the finer metallic sounds, and finally to elastic bougies. After previous dilatation with a sponge tent and the separation of the lips of the uterus with two of Sims' hooks, even those strictures situated higher become accessible to the sight, provided they are not situated too high up.

In making the diagnosis we should, however, be on our guard not to confound genuine strictures with any of the following physiological or pathological conditions, namely: the normal tone of the muscular fibres; spasmodic or inflammatory stricture; engorgement of the mucous membrane as the result of chronic catarrh; tumors, such as polypi, fibromata, ovula Nabothi; unusually deep furrows between the folds of the mucous membrane in the cervix; congenital contraction of the calibre of the cervical canal or of its orifices; cervical elongation arising from a variety of causes; concentric wrinkling of the calibre of the canal from senile atrophy; flexions of the uterus, especially from atrophy occurring at the point of flexion, etc.

The great credit is due to H. Bennet[1] of repeatedly maintaing that in healthy women, in consequence of the normal tone of the circular muscular layer, the internal os uteri is kept contracted in the same manner as the anus; though we cannot in an anatomical sense speak of a sphincter of the internal os. In consequence of this physiological tone, the point of the sound meets with a certain resistence when the attempt is made to pass the isthmus; so much the more, when it is forced onward—in its resemblance then to any foreign body—does it instantly excite a more vigorous contraction of the circular muscular fibres; which, however, at once gives way on gentle pressure with the point of the sound, by virtue of the intrinsic elasticity of the muscular layer. The absence of this resistance, in the attempt to cause the point of the sound to pass the isthmus, occurs in many pathological conditions; it may be noticed commonly, for example, in chlorosis or enlargement of the corpus uteri from various causes, such as fibromata, chronic inflammation or incomplete involution after parturition. In regard to the opinion of H. Bennet just referred to, it is evidently correct that only in rare cases, with an abnormal *exaltation* of the above-mentioned muscular contraction, should the existence of spasm, or spastic stricture, be admitted; such a condition is more frequent in extremely nervous women, or with erosions or ulcerations about the isthmus, or with flexions. In contradistinction to the normal tonicity, this spasmodic contraction manifests itself when it is only with great difficulty and severe pain that the sound passes the isthmus, by which it is felt to be tightly grasped—and this in women who have not suffered from any such affection, or

1 Braithwaite's Retrospect of Medicine. Vol. LXVII., Jan.-June, 1873, p. 305. Vol. LXVIII, July-Dec., 1873, p. 303.

been subjected to any such treatment, as could produce an actual stricture at that point.

The inflammatory strictures, which depend upon an excessive engorgement of the mucous membrane, as the result, for instance, of blennorrhœa, manifest themselves through the usual symptoms of an acute inflammation, and recede in the same proportion as the inflammation subsides. Long continued catarrhs produce engorgement of the cervical mucous membrane, which then is generally felt to be soft and flexible, in contradistinction to the condition in callous strictures. Tumors, such as polypi, fibromata, ovula Nabothi, etc., at the same time that they cause the distance between the walls of the cervical canal to become greater, also occlude its passage. They are usually accessible to the point of the index finger. With regard to the impediment offered to the sound on account of the depth of the sulci in the cervical mucous membrane, this happens only when we make use of a sound uncommonly fine. The circumstance that the ordinary button-sound can be introduced with facility, clears up any mistake. Congenital strictures of the cervical canal are met with very rarely. I have seen only one such case. It was that of a married lady, A. C., forty-eight years old, living here in Stockholm, whose vaginal portion was unusually small but conical, and the external os so contracted that only a very fine elastic sound (French scale, No. 4) could be introduced, and in the canal it was impossible with the point of the sound to make the ordinary manœuvres from side to side ("sidoexkursionerna"). The woman had always been very thin and troubled with anemia, had scant menstrual bleeding, and this explains why the dysmenorrhœal symptoms had been comparatively mild[1]. She was sterile. Neither engorgement of the mucous membrane nor any trace of a cicatrix was discovered on the margin of the os uteri, for which reason the stricture was considered to be congenital.

If the cervix is elongated through stretching produced

---

1 Courty, op. cit., p. 441.

for instance by the presence of an ovarian tumor,[1] this pathological condition explains in an unmistakable manner the diminution in the calibre of the canal. Concentric wrinkling of the calibre of the cervical canal from senile atrophy, or obliteration, of the os uteri—be it external or internal—such as occlusion resulting from ulceration or adhesion of the granulating surfaces, is seldom observed, and has principally a pathologico-anatomical interest, as the patients in general are unconscious of the existence of these alterations. Constriction of the isthmus by flexions is very rarely observed; and, we may say, exclusively in very old flexions, where atrophy has occurred at the point of flexion. In treating the ulcerative cervical catarrhs, which usually accompany flexions, through the repeated introductions of solid caustics,[2] strictures are produced of one or the other form.

PROGNOSIS.

*The Prognosis* comprises not only a consideration of the natural course of the disease, but also the prospect which the patient may have of being relieved of her affection. The disease itself constitutes a very serious lesion of the female sexual organs, depending not only upon the fact that it lies in the nature of the disease to become generally more and more aggravated, but also on the troublesome consequences which follow it, such as engorgement of the vaginal portion, hæmatocele, perimetritis, etc. A striking contrast to this view of the case is presented in the remarkable change for the better in the patient's entire constitution in short, which takes place after the operation.

---

1 [ ". . . The cervix may be drawn up out of reach, or the whole uterus may be elongated, when the connection with an ovarian tumor is close; or the lower portion of an ovarian tumor may be so moulded to the true pelvis that the uterus is pressed upwards and forwards, or flattened behind the pubes." T. SPENCER WELLS: *Diseases of the Ovaries; Their Diagnosis and Treatment.* London, 1872, p. 188.]—TRANS.

2 [Among the causes of cicatrices of the cervix, Dr. Skene mentions "destruction of the mucous membrane and subjacent structures by the free use of caustics." Cicatrices of the Cervix Uteri and Vagina. By Alex. J. C. Skene, New York. Transactions American Gynecological Society. Vol. I, 1876, p. 92.]—TRANS.

PROPHYLAXIS.

*The Prophylaxis* is the most important point. The principal rule is—in diseased conditions of the cervix, e. g., catarrh, ulcerations, etc.,—to avoid strong caustics.[1] As a rule in every uterine catarrh, whether simple, erosive, ulcerative or granular, the greatest attention should be bestowed upon the patient's general condition, and her chlorosis combatted by appropriate measures directed thereto. Local treatment may begin with a mild astringent, e. g., tannin crayons, and then if this proves insufficient, we may advance to the more vigorous, e. g., sulphate of copper, in more or less dilute solution (1:5 to 1:50); but under no circumstances ought we to make use of any agent which has been charged with the production of stricture[2]—its employment at least ought not to be re-

1 [Dr. Mary Putnam Jacobi, of New York, has made careful and valuable investigations in regard to "THE ACTION OF NITRATE OF SILVER ON EPITHELIAL AND GLAND CELLS," (*Transactions Medical Society State of New York*, 1875). These researches relate more particularly to the effects of the agent on the gastric mucous membrane, founded upon experiments on the lower animals, but have by analogy a bearing also upon its erosive action on epithelial and glandular structures in general.]—TRANSL.

2 [Dr. Robert Battey, of Rome, Ga., recommends for the local treatment of uterine disease, a combination of iodine and carbolic acid, under the name of iodized phenol, which he has successfully employed for several years, and which has been very favorably received by the profession here.

For cancerous affections of the uterus the following formula is employed:

"Recipe No. I.—Take of iodine, one half ounce; crystalized carbolic acid, one ounce. Mix and combine the two by gentle heat."

For the more frequent and less violent disorders of the uterus, the above formula is mitigated as follows:

"Recipe No. II.—Take of iodized phenol, one ounce and a half; crystalized carbolic acid, one ounce; water, two drachms. Mix and make solution."

"This preparation has been very fully tested by the writer in a large number of cases, and in a variety of uterine disorders; e. g., chronic affections of the cervix, the cervical canal and the endometrium, uterine hypertrophy and subinvolution. It has been used both in its full strength and in various degrees of dilution with glycerine; sometimes two-thirds the above strength, sometimes one-half, one-third, and even one-fourth. The strength used has been determined, first, by the mode of application proposed; second, by the energy of the effect desired; and third, by the tolerance of the patient." * * * "Of the immediate effects of this treatment, it may be said that the pain inflicted, even by the strongest application, is for the most part very trifling, and in quite numerous instances, absolutely none at all. In this respect it presents a striking contrast to the nitrate of silver. The carbolic acid, acting as a local peated. Against obstinate catarrh, or chronic blennorrhœa, the cold water cure constitutes the first remedial measure, so as to ensure hydro-therapeutic treatment, and in most cases a modified one—e. g., with cold spongings in the morning and cold sitz baths in the evening—is sufficient to inaugurate the patient's cure.[1].

---

anæsthetic, allows us to make powerful caustic applications of the iodine with little or even no pain." * * * "Whatever may have been the strength of the applications, stricture of the os and cervical canal, too often an unpleasant sequel to the use of nitrate of silver, has not resulted in any case. When applied to the cervix and cervical canal, in a caustic way, the reproduced tissue is normal and not cicatricial in character. It is believed that the very full absorption of iodine by the uterus, in this method of treatment, exerts a decidedly alterative influence over the diseased organ; and more than this, the iodine thus carried into the general circulation is highly beneficial as a constitutional remedy also. It may, therefore, be confidently asserted that iodized phenol should have a place among our topical applications to the diseased uterus."—*American Practitioner*, February, 1877.]—Translator.

1 [For two of the most concise and comprehensive brochures in the literature of the subject, on the treatment of diseases of the uterus, see "Surgery of the Cervix in Connection with the Treatment of Certain Uterine Diseases," (*American Journal of Obstetrics*, Feb. 1869), and "The Philosophy of Uterine Disease," (*New York Medical Journal*, July, 1874). By Thomas Addis Emmet, M.D., of New York.

The latter article, among other valuable teachings, contains Dr. Emmet's method of treatment by the hot water vaginal injections, which in its striking results marks an era in uterine therapeutics.

In regard to the use of caustics, Dr. Emmet here says: "Rare indeed is the necessity for applying, within the uterine canal, caustics, the cautery, or the strong mineral acids. It is true that these remedies act promptly, so far as to heal an erosion and to check all uterine discharge. But we cannot restore the patient to health by so far changing the character of the mucous membrane as to leave a mere cicatricial surface. Our ultimate success will be directly in proportion to the condition in which we leave this membrane, for we will need its healthy action in the after-treatment of the case. That individual cases escape with but little damage is only due to protection afforded by the secretions; yet the practice, as a rule, is disastrous enough to deprecate their use. We have no remedy which will act with more promptness than the nitrate of silver, when applied to the mucous membrane of the cervix, yet it has done more damage than any other. From being in common use it is the more dangerous, for its repeated action will ultimately destroy the mucous follicles, harden the tissues, and close the os as certainly as the application of the actual cautery. The evil effects of its application on the mucous membrane of other parts of the body are so well recognized, that its continued use for the uterine canal is remarkable.

"I have found most useful for applications to the cervix, Squibb's impure carbolic acid, or creosote-tar. Its action is very different from that of the pure carbolic acid; it exerts a local anæsthetic effect, and is not a caustic. This may be applied at intervals of ten days with the intermediate use of tannin and glycerine, or the pinus canadensis. It is advisable to add to the last pint of the hot water injection a certain quantity of chlorate of potash, coloride of sodium, borax, carbonate of soda, or alum, as may seem indicated."]—Translator.

TREATMENT.

The object of *treatment* is the re-establishment of the normal calibre of the cervical canal, and this is no easy problem to solve. There are principally three methods which have been employed for accomplishing this result, namely: forcible dilatation, gradual successive dilatation with elastic sounds, and incision.

With the forcible dilatation, with PRIESTLEY'S or ELLINGER'S instrument for example, I have no experience, and I should scarcely ever venture to employ this method for the reason that, on account of the inflexibility and hardness of the cervical mucous membrane, one can never determine beforehand where the rupture will stop. In regard to the second method, or gradual successive dilatation through the introduction of larger and larger elastic bougies, I can from my own experience assert that it does not accomplish the object, notwithstanding the warm advocacy of COURTY,[1] W. CUMMING,[2] M. DUNCAN[3] and others. There remains consequently only the incision, which is recommended by BARNES, SCHRŒDER, HEGAR and KALTENBACH, BEIGEL and others. But upon this I will first of all remark, that there is a difference, inexpressibly great, between the division of a stricture, which—in its resemblance to internal urethrotomy—is an easy operation, and incision of the *entire* cervix with a double-bladed hysterotome; and this seems to me a dangerous point in regard to the latter, that the veins about the internal os are very large and present the appearance of more or less rigid tubes, and besides the largest branches of the hypogastric artery enter the uterus at this point.[4]

1 Courty, op. cit., p. 447.

2 Braithwaite's Retrospect of Medicine. Vol. LXVIII., July-Dec. 1873, p. 306.

3 Ibid. Vol. LXVII., Jan.-June, 1873, p. 304.

4 [In regard to the necessity of avoiding the incision of the internal os, Dr. Robert Barnes expressed the following views at the meeting of the American Gynæcological Society, in New York: "I have known fatal results to follow the operation of incision [of the os internum]; and cellular inflammation, etc., are not rare consequences. It is unquestionably a dangerous operation, for we cannot tell how near the vessels are to the surface; moreover, they traverse a dense tissue, where, if cut, they cannot be easily controlled; finally, the incision is also favorable to the entrance of septic matter." * * * *

The method of operating, to which I give the preference, is that adopted by SCHRŒDER, because it is simple and practical. It is of essential importance that before the operation an earnest study should be bestowed upon the special case presented, which should be, as much as possible, individualized; so that the method of operating shall be accurately adapted to the previously existing condition. In the performance of the operation itself, it is well to do neither too much nor too little, but to observe the golden mean.

Not a few instruments, particularly hysterotomes, have been constructed for performing the operation as simply and easily as possible. It would be too tedious here to enumerate and give a description of them all; therefore I will confine myself to a statement of how experience has taught me we may proceed.

The patient having been placed in the side position, SIMS' speculum and a retractor are introduced, and the vaginal portion fixed with a SIMS' hook. Chloroform as a rule is not generally employed, because the operation is accomplished without the patient's being conscious of it. We should, through bimanual examination, and in the last moment through repeated pressure with the point of the index finger against the anterior and posterior roof, inform ourselves accurately as to the shape and position of the uterus.

With an obliterating stricture a straight fistula knife is introduced gently and slowly through the external os, in the direction of the canal, until we feel that the resistance is overcome. The knife is then withdrawn, and the pointed branch of KUCHENMEISTER's scissors introduced into the cervical canal, the hook of the other arm is fixed in one side of the cervix at some distance from the roof, and with a quick cut this side of the vaginal portion is divided, the scissors are then revolved a half circle on

" For division of the os externum as a means of relieving dysmennorrhœa, the incision need not be large, and need not extend so far back as the insertion of the vagina; it can easily be accomplished by a minor operation, and is most conveniently performed by means of scissors. In some cases the tissues undergo a certain amount of contraction, and the operation may need to be repeated; success, however, usually attends the first operation."—*Trans. Am. Gyn. Society*, Vol. I., 1876.]—TRANS.

their axis, and the same proceeding repeated on the other side of the vaginal portion. Should the cervix be unusually long and conical, and the cervical canal narrow also, I am in the habit then of immediately introducing a SAVAGE's hysterotome nearly to the extent of an inch into the canal, but at the same time with the greatest care avoiding the entrance of the point of the instrument within the internal os; after which a bilateral cut of the proper depth is made through that portion of the cervix which was not divided by the scissors. With cicatricial stricture merely of the external os, bilateral division with KUCHENMEISTER's scissors, or incision with MARION SIMS' knife, is sufficient.

The callous strictures, if they are of a high grade, are dilated by introducing, one after the other, larger elastic sounds, until a director can be introduced, on the groove of which the stricture is divided unilaterally, or bilaterally, with a sharp fistula knife.

The ethmoid strictures are dissected up by incising the strong fibres of connective tissue until we perceive that the resistence is overcome, when the uterine sound can be introduced with ease and make the ordinary side excursions.

As the hemorrhage has in general been so inconsiderable, and no unfavorable reaction has ever been observed after the operation, I have not thought it necessary to enter into a minute detail of all the operations which I have performed on the examination table in my consultation room—with the exception of Case VI. I will add, however, that all the operations were undertaken in the warmest season of the year, and in patients belonging to the laboring, or at least to the more tolerant, class; and whose time did not allow them unnecessarily to remain in bed a single day. During the cold season of the year and in more delicate persons, I never operate otherwise than at the patient's home. On all occasions I stand on the side of those writers who advise the very greatest circumspection. In general, I have, after operating, merely painted the surface of the wound, made by the operation, with a solution of chloride of iron by means of PLAYFAIR's sound, until the bleeding ceased, and have then introduced a tent of gossypium hæmostaticum into the canal and carefully tamponned the fundus vaginæ with boracic acid tampons, which were daily changed. Yet before this was done a solution of permanganate of potassa was injected, after which the track of the wound was opened up by manipulations with the sound. I have never introduced sponge tents after the operation. On and from the fifth day after the operation the patient has been sounded regularly twice a week with large elastic bougies (No. 18–20) or with the ordinary uterine sound.

Any troublesome consequences after the operation, such as thrombosis, oophoritis, peri- or para-metritis, septicæmia or the like, I have hitherto never experienced.

### CONCLUSIONS.

[From the foregoing, the TRANSLATOR recapitulates Dr. EKLUND's views in the following synoptical statement:—

1. That from a general review of the medical literature of various countries, strictures of the cervical canal and of the internal and external os are becoming more and more frequent every year.

2. That a variety of different causes may produce stenosis; but that, practically, it is of the greatest importance to discriminate accurately between the most frequent and certain causes, and those, on the other hand, which only very rarely exert this influence.

3. That it is entirely undemonstrated that blennorrhœa[1] is the essentially originating cause, as these strictures constitute in prostitutes—who are most subject to blennorrhœa, and to an intense degree—a very rare or else little noticed disease. And that, consequently, far less have we reason to believe that stenosis of the cervix in

1 [It will be observed that during the course of this paper the author has used the term "blennorrhœa" in its comprehensive and etymological signification—*a flow of mucus*, whether depending upon a specific virus or upon the various conditions giving rise to uterine catarrh in its various forms. Hence, it is synonymous, sometimes with gonorrhœa, and sometimes with leucorrhœa; the context, however, in each instance, rendering the meaning plain, I have preferred here, as well as in the use of other expressions, to give a literal translation, and adhere to the nomenclature employed by the author].—TRANSL.

married women is produced by gonorrhœa in their husbands, even admitting—though only exceptionally, and far from being the rule—that from a gonorrhœa in the husband originates acute blennorrhœa in the wife. Also, that many cases come under observation, both married and single, who have evidently been afflicted with blennorrhœa for ten, twenty or thirty years, without strictures occurring in the cervical canal. And that, therefore, we cannot adjudicate to gonorrhœa any special prominence among the causes of these strictures.

4. That, of the diseased conditions affecting the cervical canal, catarrh, from its frequency, is the most prominent—exhibiting different intensities, from simple catarrh, through the intermediate erosive and ulcerative forms, up to the severer forms of granular (vegetating and fungous) catarrhs. That the milder forms of uterine catarrh, even in conjunction with erosions, may recover under a proper treatment, directed merely against the general derangement; and that the ulcerations so often observed are usually healed without cicatricial contractions, if we only refrain from the use of powerful caustics: whereas, the more extensive ulcerations and granulations demand local treatment, in which we must determine to employ such agents as shall counteract, if possible, the tendency to cicatricial contractions, which the severest forms of this pathological condition necessarily occasion. That for effecting a cure of these conditions the most varied forms of caustic and astringent agents have been recommended and employed, from the ferrum candens down to the mild vegetable astringents.

5. That in the lighter forms of uterine catarrh the introduction of tannin crayons—as recommended by Prof. A. Anderson, of Stockholm—is an excellent application. That in the severer cases with ulcerations, hypertrophy and neoplasms of the papillæ, the best agent is sulphate of copper, with which in dilute form (1:5 to 1:50) the author has had extensive experience, applying it by means of an applicator to the entire interior of the uterus—it being very efficacious, without being followed by any inconvenience, such as erosion of the mucous membrane, which is produced by some of the other agents employed.

6. That, belonging to the first or most frequent class of causes, is the abuse of solid caustics—standing in the foremost rank—producing deep solutions of continuity, when employed in the treatment of pathological conditions within the cervical canal: and that, thus, all who employ caustics in daily practice, become chargeable with producing stenosis of the cervix.[1]

7. That neither can traumatism during parturition, nor the puerperal inflammations, be regarded as prominent among the producing causes. In regard to the former, although the lacerations which occur in the cervix, heal with a cicatrix, yet on account of the enormous dilatation of the cervix during parturition, the calibre of the canal afterwards shows a dilatation which is most considerable at the external os, or just at that point where the lacera-

---

1 [In further confirmation of Dr. Eklund's views, in regard to the abuse of solid caustics, I note also the following, from Dr. Emmet: "The nitrate of silver in the solid form is in more common use, from its supposed mild action, than any other agent for local treatment; yet from indiscriminate and too frequent use it has done more harm than any of the strongest caustics. I grant that its use will heal an erosion promptly and sooner, probably, than any other means we have at command, but it is accomplished at the expense of an impaired vitality of the parts. Its use I have almost entirely abandoned, and confine myself chiefly to a solution not stronger than forty grains to the ounce, to aid the action of some previous application. It is not that I would so much deprecate its use in the hands of an expert, but, from its convenient form, it is too great a temptation for many who are the most ignorant to flatter themselves that they have mastered the art as a specialty when once in possession of a porte-caustique and a speculum. This practice has become a scandal to the profession. * * * As I confine my local treatment chiefly to the canal, I do not use any of the caustics proper, for fear of contraction from cicatricial tissue; although I have observed that the tendency to its formation does not exist to the same extent within the canal, after the use of proper remedies, as on the surface of the neck."

"We are indebted, I believe, to Dr. Sims for the introduction of chromic acid in the treatment of uterine disease. It is a remedy which I have had in daily use for at least twelve years past, and my experience has been that its use is attended with less objection than from any other agent. It should not be used of a greater strength than equal parts by weight with water. Its effect then on healthy tissue is not greater than that of the strong tincture of iodine. It acts on a diseased surface as a stimulant and as an astringent, protecting it with a thin film, which usually is not thrown off from the uterine canal under a week. I am in the habit of applying it a day or two after each menstrual period, and after a week or ten days using various other remedies of a milder character as adjuvants. I think, as a rule, that we expect too rapid a result from local treatment, and are consequently tempted to resort to stronger or more prompt means than are necessary."—Emmet: Surgery of the Cervix.]—Trans.

tions were deepest and most numerous. And, that we should as little adjudge to the puerperal inflammations any prominent influence in the production of stenosis; for, as often as these inflammations are met with in the uterus and its annexa, so seldom upon them can the production of stenosis be logically predicated. That the same reasoning applies to blennorrhœa as a cause; for, as before stated, while it occurs very commonly in prostitutes, stenosis of the cervix is in this class of women extremely rare, or else little noticed. Also, that the theory that strictures are produced in consequence of rupture of the ovula Nabothi, the granulating walls becoming adherent to each other, it is quite certain we very rarely have the opportunity of observing, if ever at all.

8. That among such causes as exert this influence very rarely, perhaps should be mentioned: deep spontaneous ulcerative lesions occurring during pregnancy; likewise, diphtheritic formations, and vaginal injections with caustic liquids; besides, in the non-gravid, contracting exudations after inflammations of the inner margin of the lips of the uterus, with or without ulceration.

9. That strictures of the cervical canal and of the internal and external os may be classified and tabulated as follows:

*I.*—Obliterating Strictures.

*A. Totally Obliterating, or Adhesive in the Proper Sense.*

*B. Impermeable in the Surgical Sense.*

*a.* Adhesive Impermeable.

*b.* Ethmoid Impermeable.

*II.*—Cicatricial Strictures.

*III.*—Callous Strictures.

A. *Circular Callous.*

B. *Semi-circular Callous.*

C. *Diffused Callous.*

10. That, in making the diagnosis, we should be on our guard not to confound genuine strictures with any of the following physiological or pathological conditions, namely: the normal tone of the muscular fibres; spasmodic or inflammatory stricture; engorgement of the mucous membrane, as the result of chronic catarrh; tumors, such as polypi, fibromata, ovula Nabothi; unusually deep furrows between the folds of mucous membrane in the cervix; congenital contraction of the calibre of the cervical canal or of its orifices; cervical elongation arising from a variety of causes; concentric wrinkling of the calibre of the canal from senile atrophy; flexions of the uterus, especially from atrophy occurring at the point of flexion, etc.

11. That, as to prognosis, it is in the nature of the disease to become more and more aggravated when left to itself, and to be followed by such serious results as engorgement of the vaginal portion, hæmatocele, perimetritis, etc.; and that standing in striking contrast with this view of the case, is the prospect which the patient has of a remarkable change for the better in her entire constitution, after the operation.

12. That the prophylaxis is the most important point; the principal rule of which is: In diseased conditions of the cervix—catarrh, ulcerations, etc.—avoid the use of powerful caustics[1].

13. That, in regard to operative treatment, of the three measures employed—forcible dilation, gradual dilation, and incision—the last is the only method to be relied on or recommended.

14. That, before operating, a careful study should be made of each case presented, which should be as far as possible "*individualized*;" so that the method of operating shall be accurately adapted to the existing condition—some cases requiring simple incision of the stricture, as with Sims' knife, etc., others bilateral division of the cervix, below the external os, with the proper instruments, such as Kuchenmeister's scissors and Savage's hysterotome. That from the fifth day after the operation the patient is to be

1 [Prof. T. Gaillard Thomas, of New York, in his chapter on Dysmenorrhœa (*Practical Treatise on the Diseases of Women*, fourth edition, p. 587), says: "Contraction of the cervix may be congenital, or may result from inflammation of the mucous lining of the canal, diminution of its calibre by contraction of lymph poured out into the parenchyma, or from the use of strong caustics within the os. The last cause is a prolific one, the condition seldom failing to result from the passage of the actual cautery, or potassa cum calce, into the canal of the cervix."]—Translator.

sounded regularly twice a week with large elastic bougies (No. 18–20), or with the ordinary uterine sound.

15. That in all particulars—as to time, place, method and extent of operation, dressings, antecedent and subsequent treatment—the greatest discrimination and circumspection should be observed.

16. That after the method of treatment recommended, very satisfactory results have been obtained, and no troublesome consequences have ensued.]

